# Hypolipidemic Effect of Rice Bran Oil Extract Tocotrienol in High-Fat Diet-Induced Hyperlipidemia Zebrafish (Danio Rerio) Induced by High-Fat Diet

**DOI:** 10.3390/ijms25052954

**Published:** 2024-03-03

**Authors:** Naicheng Liu, Peng Zhang, Mingyang Xue, Mengwei Zhang, Zhenyu Huang, Chen Xu, Yan Meng, Yuding Fan, Wei Liu, Feixiang Zhang, Peng Chen, Yong Zhou

**Affiliations:** 1 Yangtze River Fisheries Research Institute, Chinese Academy of Fishery Sciences, Wuhan 430223, China; nchengliu@163.com (N.L.); somnium_zp@163.com (P.Z.); xmy@yfi.ac.cn (M.X.); zmw@webmail.hzau.edu.cn (M.Z.); hzy@yfi.ac.cn (Z.H.); xuchen@yfi.ac.cn (C.X.); mengyan@yfi.ac.cn (Y.M.); fanyd@yfi.ac.cn (Y.F.); liuwei@yfi.ac.cn (W.L.); fx3170186320@163.com (F.Z.); 2 College of Fisheries and Life, Shanghai Ocean University, Shanghai 201306, China; 3 Institute of Fishery Research of Xinjiang Uygur Autonenous Region, Urumqi 830099, China; cpeng11@sina.com

**Keywords:** rice bran oil extract, tocotrienol (T3), zebrafish, type 2 diabetes mellitus (T2DM) model, high-fat diet, blood fat

## Abstract

In recent years, the potent influence of tocotrienol (T3) on diminishing blood glucose and lipid concentrations in both *Mus musculus* (rats) and *Homo sapiens* (humans) has been established. However, the comprehensive exploration of tocotrienol’s hypolipidemic impact and the corresponding mechanisms in aquatic species remains inadequate. In this study, we established a zebrafish model of a type 2 diabetes mellitus (T2DM) model through high-fat diet administration to zebrafish. In the T2DM zebrafish, the thickness of ocular vascular walls significantly increased compared to the control group, which was mitigated after treatment with T3. Additionally, our findings demonstrate the regulatory effect of T3 on lipid metabolism, leading to the reduced synthesis and storage of adipose tissue in zebrafish. We validated the expression patterns of genes relevant to these processes using RT-qPCR. In the T2DM model, there was an almost two-fold upregulation in *pparγ* and *cyp7a1* mRNA levels, coupled with a significant downregulation in *cpt1a* mRNA (*p* < 0.01) compared to the control group. The ELISA revealed that the protein expression levels of *Pparγ* and *Rxrα* exhibited a two-fold elevation in the T2DM group relative to the control. In the T3-treated group, *Pparγ* and *Rxrα* protein expression levels consistently exhibited a two-fold decrease compared to the model group. Lipid metabolomics showed that T3 could affect the metabolic pathways of zebrafish lipid regulation, including lipid synthesis and decomposition. We provided experimental evidence that T3 could mitigate lipid accumulation in our zebrafish T2DM model. Elucidating the lipid-lowering effects of T3 could help to minimize the detrimental impacts of overfeeding in aquaculture.

## 1. Introduction

Lipids are essential nutrients that provide critical energy and fatty acids, playing a significant role in various physiological and metabolic functions [1]. In aquatic organisms, dietary lipids are recognized as vital components, supplying the necessary energy for fish growth and development and furnishing essential fatty acids and fat-soluble vitamins crucial for maintaining cellular structure and biological functions [2]. In recent years, high-lipid diets have gained popularity in aquaculture as a cost-effective strategy. However, a common issue observed in intensive aquaculture practices is the excessive deposition of lipids in the liver, leading to fatty livers among fish populations. This surplus adipose tissue alters numerous biological pathways [3] and weakens fish immunity, making the organism more susceptible to inflammatory responses [4]. It also affects fish metabolism, reducing their disease resistance [5]. Additionally, research suggests that a high lipid content in the feed can hinder largemouth bass growth (*Micropterus salmoides*), reduce liver antioxidant capabilities, and weaken immune responses [6].

While the evidence suggests that a high-quality diet can promote fish growth, this effect is largely attributed to increased fat deposition [7]. Hence, there is a pressing need to develop natural, safe, and effective feed additives that can reduce undesirable body fat and enhance feed conversion in aquaculture [8]. Plant extracts have recently gained significant attention due to their high efficiency, diverse biological activities, and minimal toxicity [9]. Throughout history, plant-derived compounds, or phytocompounds, have been used to prevent or treat a wide range of diseases in both mammals and fish [10,11]. The tetrameric configurations of tocotrienols (α, β, γ, and δ) belong to vitamin E [12]. Tocotrienols distinguish themselves from tocopherols by their unsaturated isoprenoid side chain with three double bonds, potentially allowing for a better penetration into tissues rich in fatty layers, such as the brain and liver [13].

Protecting the liver of cultured fish in aquaculture is a prerequisite for improving aquaculture income, and reducing the deposition of lipids in blood vessels will also increase the survival rate of fish. T3 (tocotrienol) has demonstrated its effectiveness in reducing fat deposition in rats and influencing their lipid metabolism [14]. T3 successfully alleviated the negative effects induced by TNF-α on MCP-1, IL-6, and adiponectin secretion, as well as on MCP-1, IL-6, adiponectin, and PPARγ mRNA expression. Additionally, T3 inhibited TNF-α-induced IκB-α phosphorylation and the activation of the nuclear factor-κB (NF-κB). Moreover, T3 can potentially lower the risk of atherosclerosis in humans [15]. At present, the composition of the human diet has an excessive intake of sugar and lipid. Studies have found that T3 reduces the risk of atherosclerosis by reducing the synthesis of human fat. On the one hand, by studying the lipid-lowering effect of T3 in aquaculture, the benefits of aquaculture can be improved. On the other hand, the proportion of lipids in bottom aquatic products can be reduced to ensure human health.

Given China’s significant agricultural production, there is an abundance of T3 in waste materials from grain processing. T3 was selected as the research target as, on the one hand, it can make full use of agricultural waste and, on the other hand, it can help the development of aquaculture. Determining the optimal quantity and methodology for incorporating T3 additives into aquatic feed could enhance aquaculture profitability while promoting resource conservation and improved resource utilization through T3 extraction from grain processing byproducts. This discovery provides scientific guidance for the comprehensive development of T3 products.

## 2. Results

### 2.1. T3 Safe Concentration (MNLC) Test

Zebrafish embryos at 7 days post-fertilization were exposed to six varying concentrations of bezafibrate (serving as the positive control) and tocotrienol to evaluate embryo toxicity. Mortality rates and morphological abnormalities were monitored at 24, 48, 72, and 96 h. No instances of death or deformity were observed in the zebrafish subjected to tocotrienol or in the control group (Supplementary Materials Figure S1).

#### 2.1.1. Effects of T3 on Reducing Triglycerides and Cholesterol in Zebrafish Larvae

**Figure 1:**
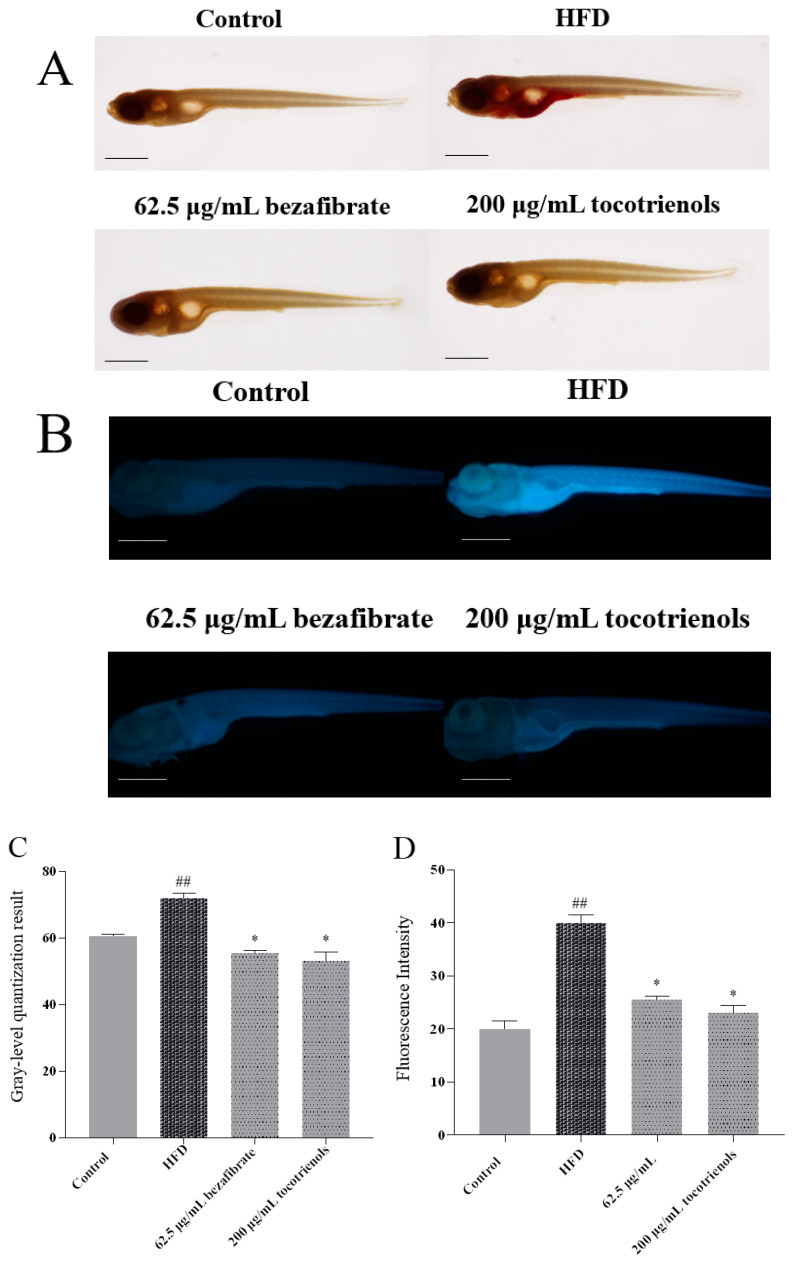
Tocotrienol can reduce the triglyceride and cholesterol contents in zebrafish larvae. (**A**) Oil red O staining results. Scale: 1 mm. (**B**) Filipin staining results. Scale: 1 mm. (**C**) Quantitative results of the oil red O staining experiment. (**D**) Quantitative results of the Filipin staining experiment. ## represents *p* < 0.01 compared to the control group and * represents *p* < 0.05 compared to the HFD group.

To assess the impact of T3 on lowering triglyceride and cholesterol levels in zebrafish, we applied both oil red O staining and Filipin staining to the following groups: blank control group, T2MD model group (exposed to high fat and 3% glucose treatment), positive control group, and experimental group. Compared to the blank control group, the T2MD model (HFD) group exhibited a significant increase in the triglyceride and cholesterol contents, with extensive deposition observed in the viscera, intestine, and heart aorta (Figure 1A,B). Filipin staining indicated a substantial enhancement in fluorescence intensity in the HFD group. The positive control drug, bezafibrate, significantly alleviated the accumulation of triglycerides and cholesterol, albeit with some remaining minor triglyceride deposition (Figure 1A,B). Following the tocotrienol treatment, no significant accumulation of triglycerides was detected in the abdomen of the zebrafish (Figure 1A), and the fluorescence intensity of cholesterol staining decreased, though some local fluorescence remained (Figure 1B). In the T3 group, the zebrafish showed significantly reduced triglyceride deposition in the heart compared to the positive control group. We found that the cholesterol content in the HFD group was significantly higher than in the control group. The addition of the positive control drug and tocotrienol reduced the cholesterol content in the zebrafish to some extent (Figure 1C,D). Additionally, a significantly higher triglyceride content was observed in the model group than in the control group. The experimental findings indicate a significant reduction in the triglyceride content in the positive control and tocotrienol experimental groups (*p* < 0.01).

#### 2.1.2. Detection of Triglycerides and Cholesterol in the Liver of Adult Zebrafish

**Figure 2:**
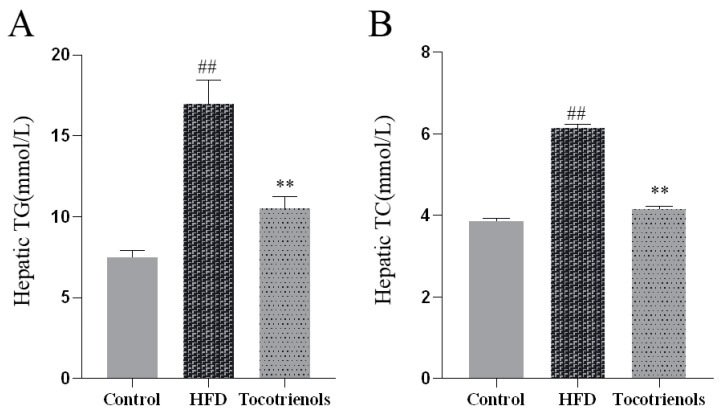
Tocotrienol can reduce the contents of triglyceride and cholesterol in the liver of adult zebrafish. (**A**) The content of triglycerides in the liver of adult zebrafish. (**B**) Cholesterol content in the liver of adult zebrafish. Compared to the control group, ## means *p* < 0.01; compared to the HFD group, ** means *p* < 0.05.

We evaluated the levels of triglycerides and cholesterol in the liver of the adult zebrafish in the T2MD model. There was a significant increase in the total serum cholesterol and triglycerides in the zebrafish administered the HFD compared to the controls (Figure 2A,B), with both surging by over 90%. However, post-tocotrienol intervention, we observed a significant drop in the triglyceride and cholesterol contents in the liver of the adult zebrafish (Figure 2A,B), decreasing by around 70%. Therefore, the experimental data suggest that tocotrienol can lower the triglyceride and cholesterol concentrations in the adult zebrafish’s liver, confirming the successful establishment of the T2MD model and making the liver suitable for the subsequent lipid metabolism analysis.

### 2.2. Vascular Thickness of the Lens Vascular System in the Eye of Zebrafish Larvae

**Figure 3:**
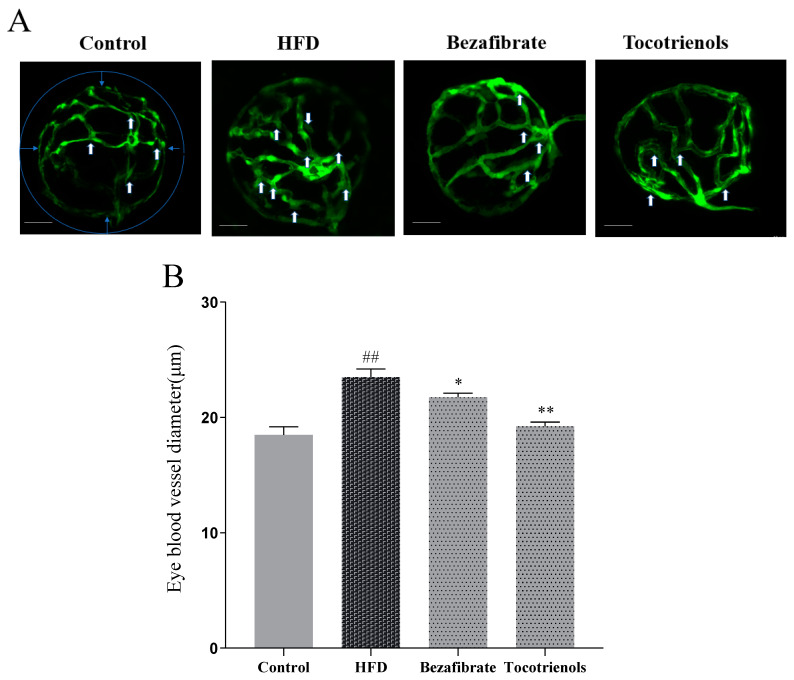
Changes in intraocular blood vessels in the zebrafish lens. (**A**) Confocal scanning of isolated Tg (fli1: EGFP) zebrafish eye, showing intraocular blood vessels in the lens. Transparent vessels can be seen from the outside through the translucent lens (indicated by white arrows in **A**), and the diameter of the intraocular blood vessels to be measured is indicated by blue arrows. Scale bar: 10 μm. (**B**) Quantification of the diameter of intraocular blood vessels in confocal scanning images, n = 6. ## indicates comparison with the blank control group, with *p* < 0.01. * indicates comparison with the model group, with *p* < 0.05. ** indicates a significant difference compared to the model group, with *p* < 0.01.

The retinal blood vessels of the zebrafish lens form a basket-like structure known as the inner circle of vision (IOC) [16]. Hyperlipidemia, characterized by elevated cholesterol and lipid levels in zebrafish blood, tends to result in lipid deposition on vascular walls, leading to IOC wall thickening [17]. In the zebrafish T2MD model, monitoring lens intraocular blood vessel diameters can assist in evaluating zebrafish blood cholesterol and lipid level alterations. Figure 3A demonstrates a substantial increase in blood vessel branching (marked by white arrows) in the HFD group relative to the control group. The administration of the lipid-lowering medication benzafibrate resulted in a relative decrease in branching, while the treatment with the phytoestrogen resveratrol significantly diminished branching. The subsequent measurements of blood vessel diameters at points denoted by the blue circle were analyzed. The data analysis revealed a notable increase in the blood vessel diameter in the HFD group relative to the control group. Moreover, T3 supplementation significantly reduced the blood vessel diameter compared to the HFD group (Figure 3B).

### 2.3. Protein Expression Levels of Pparγ and Rxrα in Adult Zebrafish

**Figure 4:**
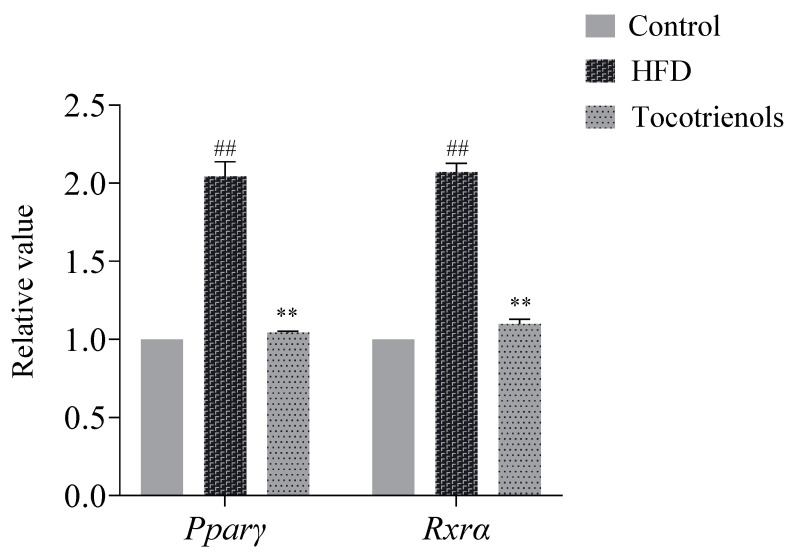
The expression levels of the *PPARγ* and *RXRα* proteins in the adult zebrafish T2MD model. the expression levels of the *PPARγ* and *RXRα* proteins in the model group were significantly higher than those in the blank group, and the expression levels of the *PPARγ* and *RXRα* proteins in the experimental group were significantly lower than those in the model group. ## indicates that, compared to the blank control group, *p* < 0.01; ** indicates that, compared to the model group, *p* < 0.01.

During the process of lipid regulation in zebrafish, the downregulation of *Pparγ* and *Rxrα* positively impacted zebrafish hyperlipidemia. In the present study, we detected the levels of *Pparγ* and *Rxrα* proteins (Figure 4). Compared to the control group, the expression of *Pparγ* and *Rxrα* significantly increased in the HFD group (*p* < 0.01), exhibiting an over two-fold increase. In the experimental group, with the addition of T3, the protein expression levels of *Pparγ* and *Rxrα* showed a significant decrease compared to the HFD group (*p* < 0.01), by nearly 50%. The expression levels of *Pparγ* and *Rxrα* in the experimental group were suppressed to the levels of the blank control group, indicating that T3 inhibited the expression of *Pparγ* and *Rxrα*. T3 may reduce the blood lipid level in zebrafish by inhibiting the expression of the Pparγ and Rxrα proteins.

### 2.4. Effects of T3 on the mRNA Expression of Lipid Metabolism-Related Genes in the Liver of Adult Zebrafish

**Figure 5:**
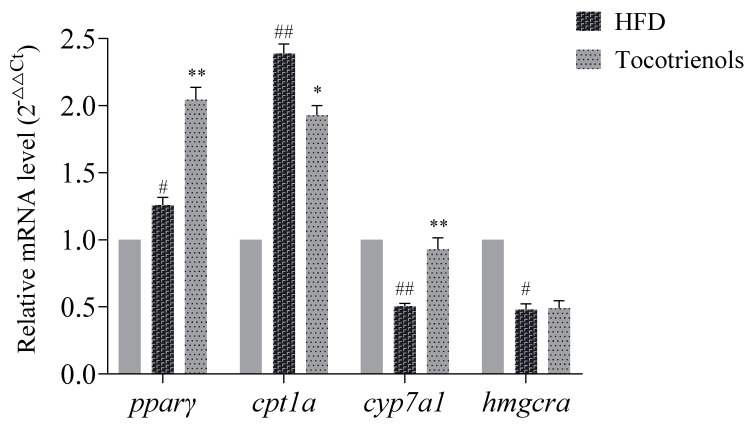
Liver lipid metabolism-related gene mRNA expression. # indicates that, compared to the blank control group, *p* < 0.05; ## indicates that, compared to the blank control group, *p* < 0.01; * indicates that, compared to the model group, *p* < 0.05; ** indicates that, compared to the model group, *p* < 0.01.

In order to explore the process of T3-regulating lipids at the gene level, the expression of liver lipid-related genes was detected by RT-qPCR. As shown in Figure 5, compared to the control group, the expression of *cpt1a* and *pparγ* mRNA in the liver of the zebrafish in the model group was significantly increased (*p* < 0.05), and the expression of *cyp7a1* and *hmgcra* mRNA was significantly decreased (*p* < 0.05). Compared to the model group, the expression of *pparγ* and *cyp7a1* mRNA in the liver of the zebrafish in the experimental group was significantly increased (*p* < 0.01), and the expression of *cpt1a* mRNA was significantly decreased (*p* < 0.05). Although the expression of *hmgcra* mRNA was changed, but there was no significant difference.

#### 2.4.1. Lipid Analysis of the Adult Zebrafish Liver

**Figure 6:**
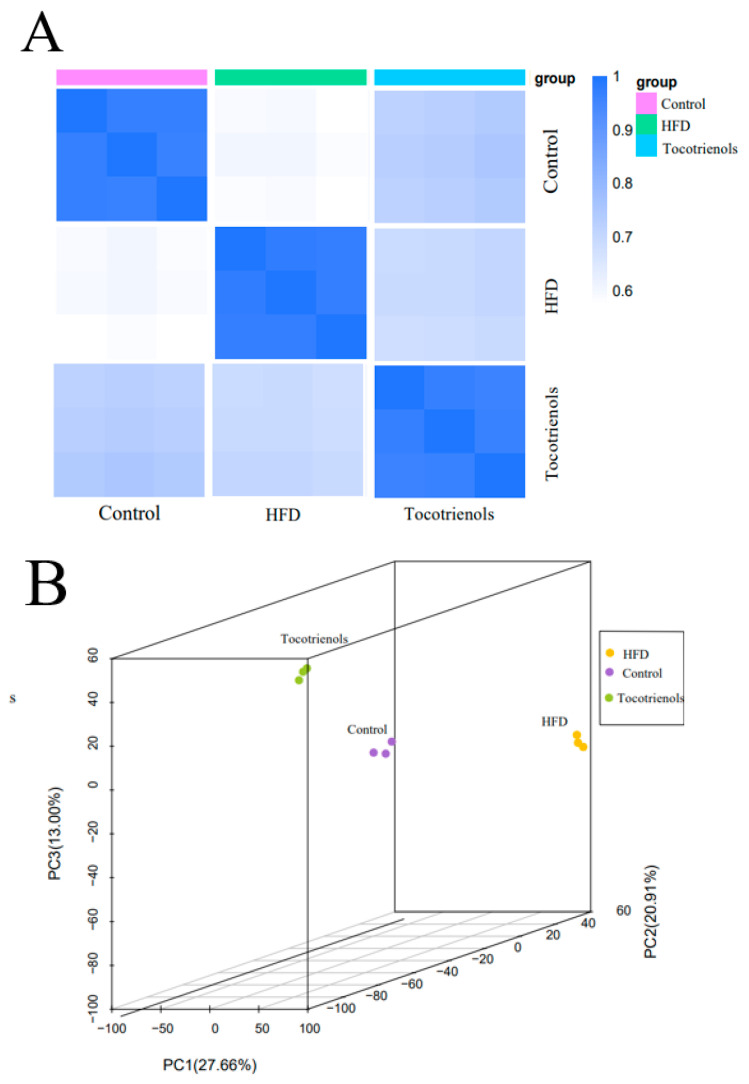

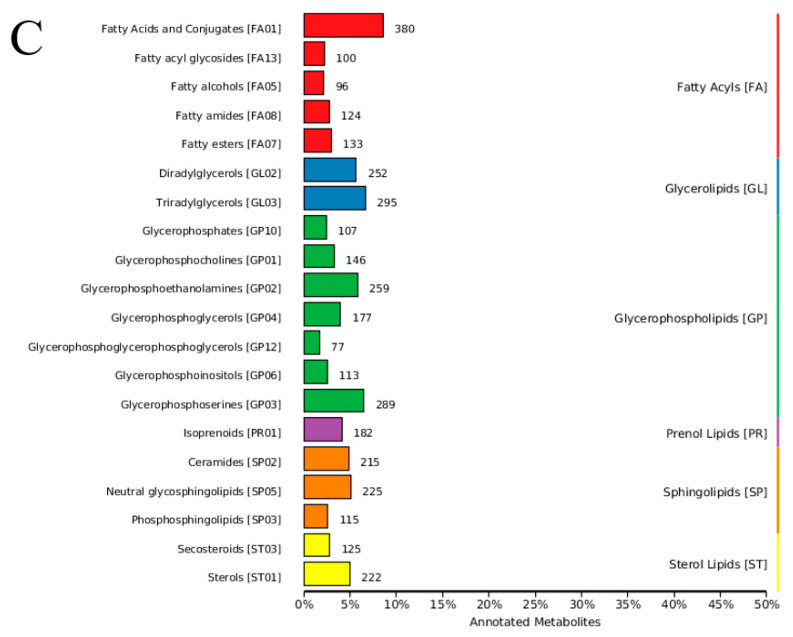
Results of the lipid analysis in the adult zebrafish liver. (**A**) Correlation analysis among the control group, HFD group, and tocotrienol group. (**B**) PCA analysis results, where the *x*-axis represents the first principal component and the *y*-axis represents the second principal component. (**C**) The TOP20 detected lipid classification results.

A metabolomic examination was executed to scrutinize the liver lipid profiles in the zebrafish. Quality control was implemented for the HFD, Control, and Tocotrienol groups. Figure 6A exhibits the evaluation of replicate correlation, employed to appraise biological replication within each group by examining correlations between samples. The data’s representativeness for the HFD, Control, and Tocotrienol groups was studied using a principal component analysis (PCA), a multidimensional statistical method, as shown in Figure 6B. The Kyoto Encyclopedia of Genes and Genomes (KEGG) database was used for lipid data annotation, identifying the top 20 pathways (Figure 6C). The top 20 most annotated pathways among the HFD, Control, and Tocotrienol groups included cysteine and methionine metabolism, lysine degradation, death by iron bile secretion, FcγR-mediated phagocytosis, arachidonic acid metabolism, unsaturated fatty acid biosynthesis, primary bile acid biosynthesis, sphingolipid metabolism, steroid biosynthesis, steroid hormone biosynthesis, alpha-linolenic acid metabolism, ubiquinone and other terpenoid-quinone biosynthesis, insect hormone biosynthesis, terpenoid backbone biosynthesis, the modulation of inflammatory mediators on TRP channels, apelin signaling pathway, phospholipase D signaling pathway, sphingolipid signaling pathway, and neuroactive ligand–receptor interactions. Notable metabolite alterations were detected in the High Carbohydrate and Fat (HCF) and Tocotrienol cohorts compared to the control group (BC) for the zebrafish.

#### 2.4.2. Comparative Analysis of the Liver Lipid Differences between the HFD Group and T3 Group

**Figure 7:**
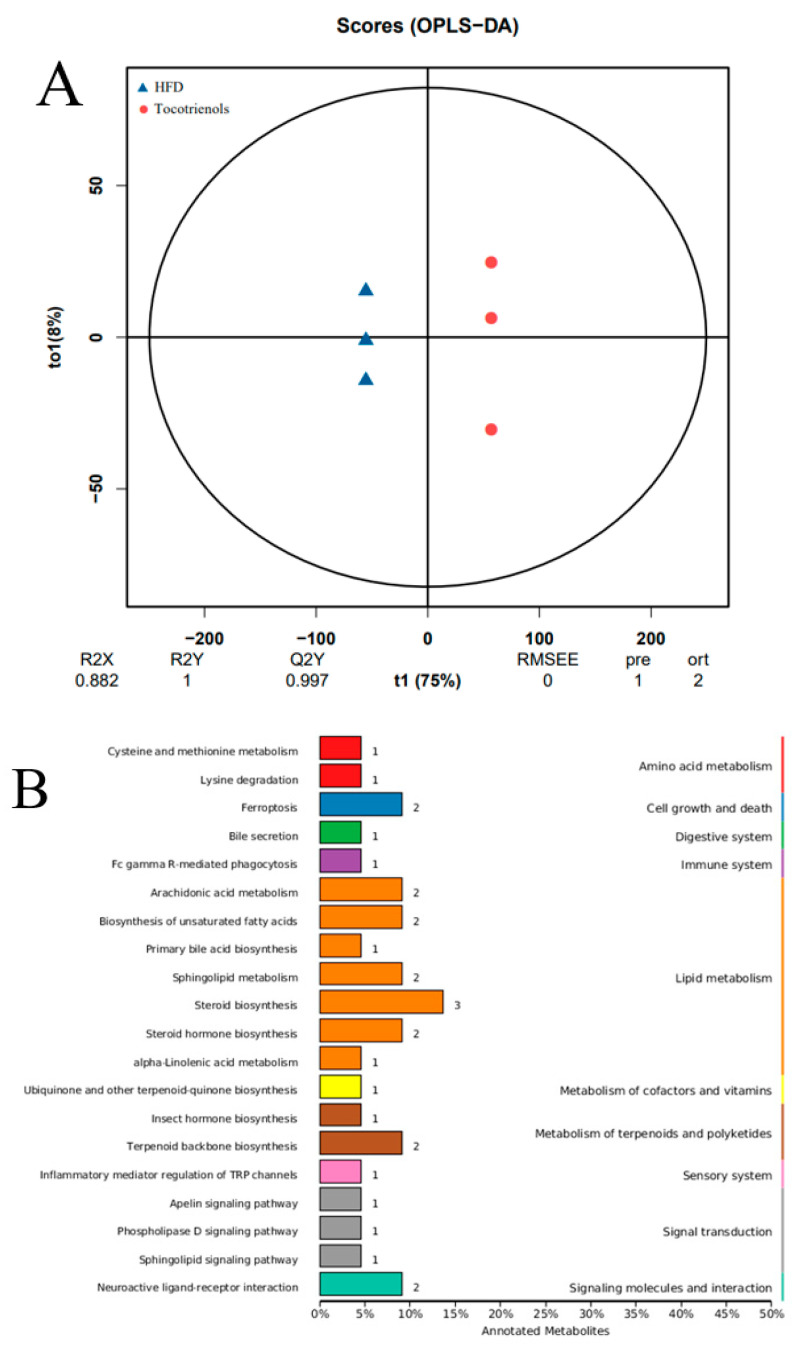

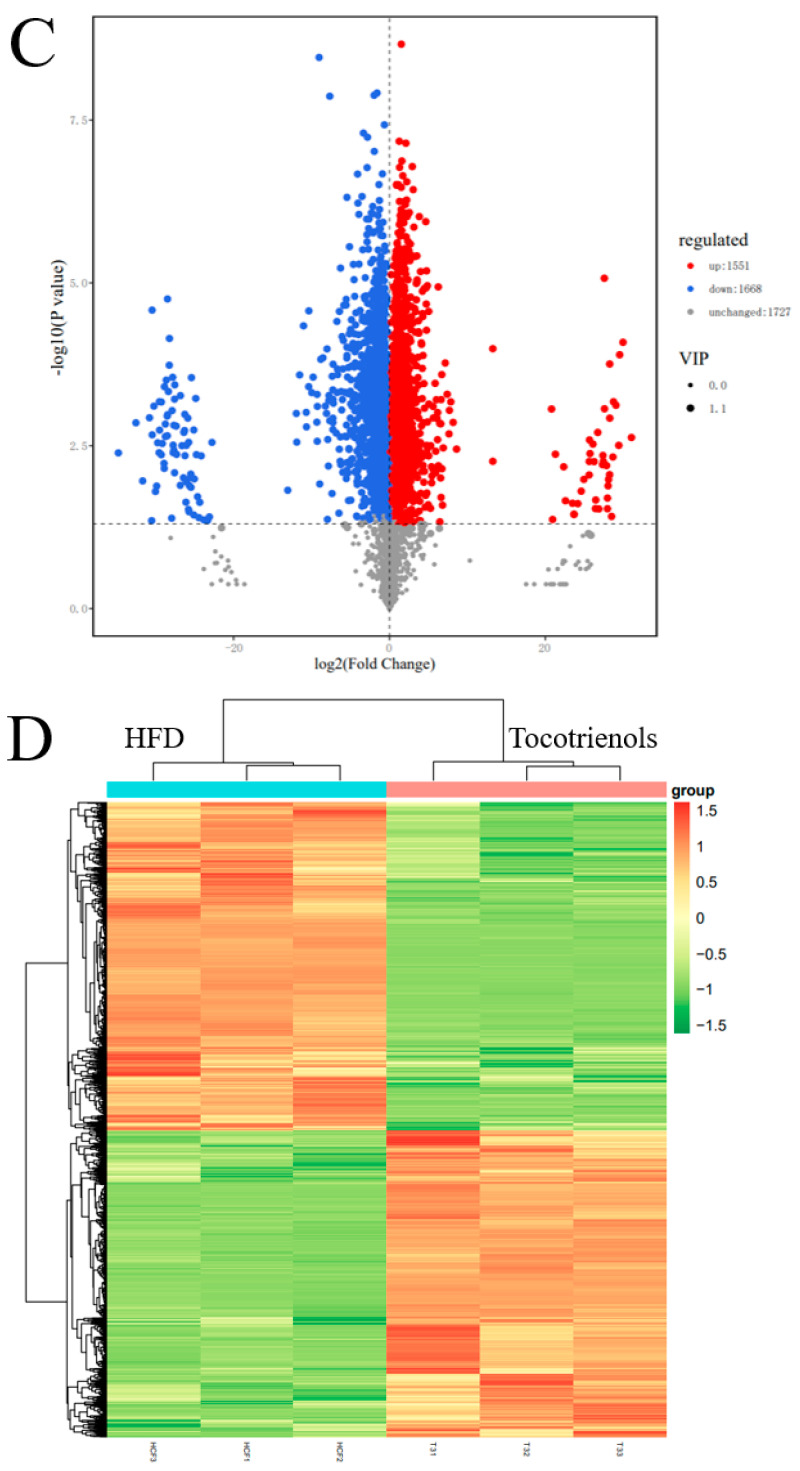
Differential lipid analysis between the HFD group and tocotrienol group. (**A**) OPLS-DA score plot. (**B**) TOP20 differential lipid KEGG annotation results. (**C**) Volcano plot results. (**D**) Differential lipid clustering heat map.

To explore the hypoglycemic and hypolipidemic mechanisms of T3 in zebrafish, we subsequently analyzed two cohorts: the HFD and the T3-treated groups. The application of orthogonal projections to latent structures-discriminant analysis (OPLS-DA), akin to PCA, served to evaluate the influence on lipid metabolism. The R2Y and Q2Y values, which assess the model’s stability and reliability, approached 1 as the model became more stable. Differential lipids were screened using this model. The resulting analysis yielded a Q2Y value of 0.995 and an R2Y value of 1 (Figure 7A), reinforcing the experimental model’s stability and reliability. The factors of relatively higher proportions in both the HFD and T3 groups encompassed FcγR-mediated phagocytosis, primary bile acid synthesis, insect hormone synthesis, the regulation of TRP channels by inflammatory mediators, apelin signaling pathway, and phospholipase D signaling pathway. The PCA (Figure 6B) and OPLS-DA (Figure 7A) plots revealed the clear separation of the HFD and T3 cohorts, indicating substantial alterations in lipid metabolite levels in the T3 group relative to the HFD group.

**Figure 8:**
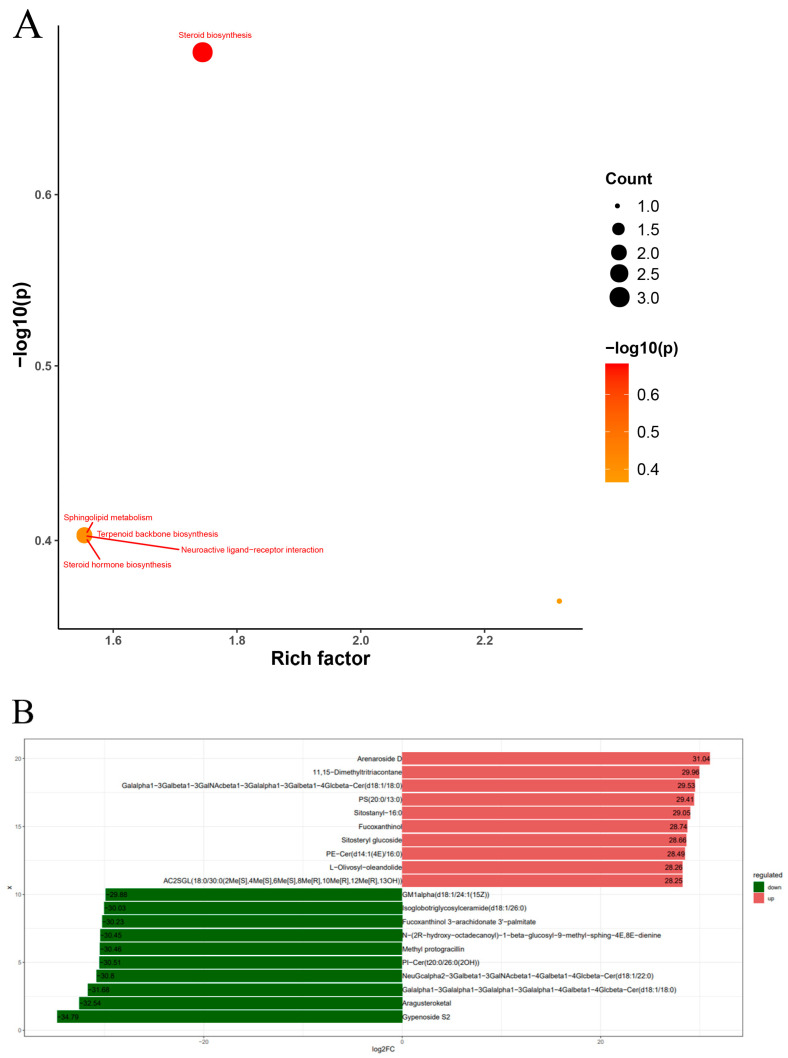
(**A**) Differential lipid KEGG enrichment factor bubble diagram. (**B**) Difference multiple histograms.

Upon performing qualitative and quantitative assessments on the identified lipids (Figure 7B), significantly enriched KEGG pathways for the differential lipids between the HFD and T3 groups included steroid biosynthesis, sphingolipid metabolism, terpenoid backbone biosynthesis, neuroactive ligand–receptor interaction, and steroid hormone biosynthesis. When comparing the HFD and T3 groups, it was revealed that the T3 group had 1668 upregulated metabolites and 1551 downregulated metabolites, as shown in the Volcano plot (Figure 7C). A hierarchical clustering heatmap was constructed to illustrate the clustering of differential lipid groups across the two comparative sets alongside a clustered heatmap devoid of substance names (Figure 7D). The top ten changes in lipid annotation based on the LIPID MAPS database are displayed in Figure 8A. Through the annotation of lipid species, a bubble plot of enriched KEGG pathways for differential lipids was derived (Figure 8B). It was found that the significantly upregulated metabolites were associated with glycerophospholipids, sterol lipids, prenol lipids, and saccharolipids (Figure 8A). These findings suggest that the T3 treatment markedly upregulates glycerophospholipid metabolites and downregulates sphingolipid metabolites. Additionally, logarithmic transformations were used to analyze the fold changes in differential metabolites. Figure 8B shows the top 10 upregulated and downregulated lipid metabolites in the experimental group compared to the control group, with the results displayed as logarithmic fold changes. These primarily comprise glycerophospholipids, sterol lipids, polycyclic ketones, sphingolipids, fatty acyls, and glycerol lipids.

The differential lipid metabolites were explored through a KEGG pathway analysis and pathway-centric network analysis. Using the KEGG database, annotations were made for the differential lipids, and the top 20 pathways containing the highest count of annotated differential lipids were selected. It was determined that these differential lipids primarily operated within the lipid metabolism, with a particular involvement in steroid biosynthesis and steroid hormone biosynthesis pathways. The KEGG pathway enrichment analysis, stemming from the differential lipid metabolites, revealed a close relationship between steroid biosynthesis, terpenoid backbone biosynthesis, and ubiquinone alongside other terpenoid-quinone biosynthesis, which implies that certain differential metabolites might concurrently influence multiple metabolic pathways. A comparison of the T3 group to the HFD group revealed that triiodothyronine could potentially shape lipid metabolism pathways, particularly impacting steroid biosynthesis and steroid hormone biosynthesis in zebrafish.

## 3. Discussion

High-lipid diets in aquaculture have experienced a notable upsurge in recent years owing to their cost-effective advantages in farming practices [18]. However, such diets can induce various metabolic stresses in farmed aquatic organisms, compromising their overall health [19,20]. It is imperative to recognize that an excessive fat content in the diet can inhibit pancreatic lipase activity and lead to detrimental hepatic fat deposition in lean juvenile red seabream (*Pagrus major*) [21]. Furthermore, a high-fat diet can disrupt the gut microbiota of Nile tilapia (*Oreochromis niloticus*) and undermine the therapeutic efficacy of antibiotics [22]. The consumption of high-starch diets has been associated with oxidative stress, inflammation, lipid metabolism disorders, and even metabolic lipid disorders, significantly hampering the growth performance of largemouth bass (*Micropterus salmoides*) [23]. Therefore, exploring new, safer, and more effective approaches to reduce fat deposition in aquaculture is imperative. The currently available evidence suggests that T3 can normalize blood glucose, triglyceride, and cholesterol levels in zebrafish with T2MD characterized by high-glucose and high-fat conditions. Moreover, T3 has ameliorated insulin resistance in zebrafish [24]. The influence of tocotrienol on aquatic organisms has been examined in the context of aquaculture, offering a sustainable and efficient feed additive that presents a novel and environmentally friendly approach to healthy cultivation.

In this study, we assessed the effects of T3 on the lipid metabolism in both juvenile and adult zebrafish using a T2MD model. In the juvenile group, the treatment with a high concentration of T3 (200 μg/mL) significantly reduced triglyceride and cholesterol levels in T2MD zebrafish. To ensure practical applicability in production settings, a lower concentration of T3 (50 μg/mL) was employed in the adult zebrafish T2MD model. Even at a lower concentration, T3 substantially decreased the triglyceride and cholesterol levels, highlighting its effectiveness in lowering lipids.

Our research aimed to investigate the hypolipidemic potential of T3 using a zebrafish T2MD model. As demonstrated by oil red O and Filipin staining, T3 effectively reduced both triglyceride (TG) and cholesterol (CHO) levels. Previous studies in humans and mice have shown that the downregulation of the *cpt1a* gene can influence the synthesis of conjugated fatty acids or polyphenols, leading to reduced plasma TG levels. Similarly, in a murine Type 2 diabetes model, a decrease in *cpt1a* gene expression decreased lipid synthesis [25]. Additionally, the hypolipidemic effect of a green tea extract was associated with the upregulation of the *cyp7a1* gene [26]. In the present study, after the T3 treatment of the T2MD zebrafish, we observed changes in the expression levels of *cpt1a* and *cyp7a1* genes in the liver, consistent with these findings. *Cpt1a* is a crucial enzyme for fatty acid oxidation located in the outer mitochondrial membrane [27]. *Cyp7a1*, an enzyme integral to the primary stage of bile acid synthesis, regulates the production of primary bile acids [28]. The cholesterol 7α-hydroxylase enzyme, encoded by the *cyp7a1* gene, plays a key role in the breakdown of cholesterol and the synthesis of cholic acid [29]. Therefore, the downregulation of *cpt1a* and the upregulation of *cyp7a1* are essential for modulating lipid metabolism in zebrafish. Notably, T3’s cholesterol-lowering efficacy in mice is believed to result from its inhibitory action on *hmgcra*, a key enzyme in the cholesterol synthesis pathway [30]. However, our study did not observe significant changes in *hmgcra* gene expression between the experimental and model groups, which may indicate that T3’s inhibitory action on *hmgcra* occurs at the enzyme level or within the zebrafish pathway, and further experiments are needed to pinpoint T3’s precise regulatory target.

*Pparα* and *Rxrα* play pivotal roles in lipid regulation [31]. Our investigation revealed that *Pparα*, when activated, formed a complex with *Rxrα* and bound to the regulatory elements of *cyp7a1* (PPRE), thus governing *cyp7a1* transcription [32]. Within the *Pparα*/*Rxrα* pathway, these two entities form a heterodimer that interacts with DNA to regulate gene transcription [31]. T3 exhibited a diminishing effect on the expression of *Pparγ* and *Rxrα*. Given that *Pparγ* critically influences fat synthesis and transport [33], the modulations in *Pparγ* and *Rxrα* induced by T3 are presumed to be primarily responsible for the observed alterations in lipid metabolism. In a rat model exposed to a high-fat diet, hepatic *Pparγ* and *Rxrα* levels doubled [34]. Similarly, exposure to bisphenol S (BPS) or overfeeding in zebrafish resulted in visceral fat accumulation and nearly two-fold increases in *Rxrα* expression [35]. In our study, the lipid accumulation observed in the HFD group was accompanied by the increased expression of these two proteins. Interestingly, in the T3 treatment group, both cholesterol and triglycerides were significantly lower than in the HFD group, and the expression levels of these two proteins were markedly reduced, suggesting that T3 may reduce lipids through its influence on these two proteins. However, further experiments are warranted to confirm this mechanism.

As a critical organ, the liver plays a central role in various metabolic activities, including regulating the lipid metabolism and glucose homeostasis [36]. To gain in-depth insights into the specific metabolites influenced by the T3 treatment and that contribute to reduced lipid levels, this study employed untargeted and targeted mass spectrometry approaches to conduct the metabolomic profiling of differentially expressed metabolites. The lipidomic analysis of zebrafish revealed that T3’s impact on the lipid metabolism primarily targeted the lipid metabolism and signal transduction pathways. In contrast to the HFD group, the biological functions of steroid biosynthesis were upregulated, and steroid hormone biosynthesis was downregulated in the tocotrienol group, attributed to T3’s impact on various metabolites, which exhibit diverse biological functions, contributing to these results. Future studies are warranted to further investigate the specific causes of these alterations through additional cohort and functional analyses. In the T3 group, the biosynthesis of cutin, suberin, and wax, glycerophospholipid metabolism, primary bile acid biosynthesis, sphingolipid metabolism, apelin signaling pathway, calcium signaling pathway, and phospholipase D signaling pathway were upregulated, while the sphingolipid signaling pathway was primarily downregulated. Glycerophospholipids, fundamental constituents of cell membranes, play significant roles in cell signaling, membrane anchoring, and substrate transport.

Sphingolipid biosynthetic pathways, found in all eukaryotes, regulate the production of membrane structural components [37]. Arachidonic acid, metabolized into pro-inflammatory eicosanoids during an inflammatory response, is recognized as an inflammatory mediator [38]. Furthermore, type 2 diabetes is categorized as an inflammatory disease [39]. Given our previous results demonstrating tocotrienol’s anti-inflammatory capacity, it can be inferred that tocotrienol may exert an anti-inflammatory function by reducing the metabolism of arachidonic acid [4]. It is worth noting that lipid deposition can increase sphingolipids [40]. However, the impact of tocotrienol on the metabolic pathways of lipids, cholesterol, and carbohydrates, as gleaned from the study of lipid metabolism-associated genes, does not fully elucidate the pathological mechanism. Indeed, as molecular biology technology advances, more transgenic zebrafish or advanced technologies for research will become increasingly feasible.

## 4. Materials and Methods

### 4.1. Zebrafish Experimental Conditions and T3 Maximal Non-Lethal Concentration (MNLC) Test 

Wild-type zebrafish (AB strain) and Tg (*fli1*: *EGFP*) transgenic zebrafish were purchased from the Institute of Hydrobiology of the Chinese Academy of Sciences, China Zebrafish Resource Center, Wuhan, China. The zebrafish were kept and handled according to *The Zebrafish Book* (zfin.org). A 1:1 ratio of male and female zebrafish was placed inside the same mating tank overnight. The zebrafish eggs were collected after spawning, examined microscopically, and cultured in an E3 medium at 28 °C. During the subsequent experiments, zebrafish at 3 days after fertilization (dpf) were selected as the experimental subjects every 24 h. All animal experiments were approved by the Animal Experimental Ethical Inspection of Laboratory Animal Centre, Yangtze River Fisheries Research Institute, Chinese Academy of Fishery Sciences (Approval ID Number: YFI 2022–zhouyong–1202).

To determine the maximum non-lethal concentration (MNLC) of bezafibrate and tocotrienols, 7-day- post-fertilization (7 dpf) zebrafish were treated with the testing drugs in 12-well culture plates (20 larvae/well) for 48 h, and the mortality was recorded at the end of the treatment. Under a dissecting stereomicroscope, dead zebrafish were identified as those without a heartbeat. In the initial test, six concentrations (31.25 μg/mL, 62.5 μg/mL, 125 μg/mL, 250 μg/mL, 500 μg/mL, and 1000 μg/mL) were used for the zebrafish embryo toxicity test for bezafibrate and tocotrienols. The specific steps were as follows: Fertilized zebrafish eggs (AB strain) were obtained by natural spawning. At least 15 zebrafish embryos per well were placed in 24-well microplates, incubated until 6 days post-fertilization, and randomly divided into five groups. The blank control group and the experimental group were set up. For each concentration gradient, there were three replicates, and each experiment was repeated three times. The mortality and malformation of embryos per hole were counted at 24 h, 48 h, 72 h, and 96 h after exposure.

### 4.2. Main Reagents

Glucose, egg yolk powder, and cholesterol were purchased from Beijing Tianyuan Co., Ltd. (Beijing, China). The bezafibrate tested were purchased from Sigma-Aldrich (St. Louis, MO, USA). The rice bran oil extract tocotrienol was provided by Yichun Turtle Life Science Co., Ltd., Yichun, China, (light yellow powder). Stock solutions of the ELLSA Kit (Jianglai Biological Technology, Shanghai, China) were prepared in 100% DMSO. The control zebrafish were treated with the carrier DMSO. The blank control group was used to prove that the solvent did not harm the larval zebrafish. During the study, no ammonia was detected in the water. The photoperiod was 14 h:10 h light/dark and the dissolved oxygen in the water was maintained at 5–10 mg/L.

### 4.3. Construction and Verification of the Zebrafish T2MD Model

For the preparation of an HFD (high-fat diet) for adult zebrafish, 10 g of egg yolk powder, 10 g of cholesterol, and 3 g of glucose were dissolved, amalgamated with 100 g of zebrafish feed, stirred homogeneously on a magnetic stirrer, and subsequently freeze-dried overnight. In the experimental group, 50 mg of tocotrienol was incorporated into the HFD. The high-fat diet for the zebrafish larvae was prepared by dissolving 10 g of egg yolk powder and 1 g of cholesterol, stirring the mixture until uniform with a magnetic stirrer, and freeze-drying overnight. An adult zebrafish T2MD model was established by feeding the zebrafish twice daily with the HFD for one month. Subsequently, the liver was harvested to measure the cholesterol, glucose, and triglyceride contents. For the zebrafish larvae T2MD model, 5 dpf zebrafish decolored with PTU were selected. The zebrafish larvae were fed the HFD twice daily for five days, each feeding accompanied by a soak in a 3% glucose solution for an hour. Following each feeding, the fresh E3 culture medium was replaced. The experimental group received 200 μg/mL of tocotrienol, the positive control group was supplemented with 62.5 μg/mL of bezafibrate, while the control group was administered 1% DMSO.

An ELISA kit was used to detect the relative expression levels of Pparγ and Rxrα protein in zebrafish adult liver induced by high glucose and high fat. The required plates were prepared first, marked as standard wells and sample wells, and 50 μL of standard products with different concentrations were added to each standard well, while 50 μL of the test sample was added to the sample wells. The plate membrane was used to seal the plate, and it was incubated at 37 °C for 30 min. Then, each well was filled with the washing solution, and after standing for 1 min, the washing solution was discarded, and the remaining liquid was removed with absorbent paper, repeating this process 5 times. Subsequently, the ELISA plates were incubated with detection antibody solutions (100 µL), HRP-conjugated antibodies (100 µL), and A and B substrates (100 µL). Following incubation at 37 °C for 15 min, 50 µL of the stop solution was added to each well. The optical density (OD) was measured at a wavelength of 450 nm.

### 4.4. Effects of T3 on Reducing Triglycerides, Cholesterol, and Vessel Wall Thickness in Zebrafish Larvae

Zebrafish embryos of the same batch developed to 5 dpf were selected in the experiment. A blank control group (2% DMSO), a model group (HFD + 3% glucose), a positive control group (HFD + 3% glucose + 0.0625 mg/mL bezafibrate), and an experimental group (HFD + 3% glucose + 0.2 mg/mL tocotrienol) were set up. Each group had 3 replicates with 10 embryos per replicate. The samples were fixed at 9 dpf and observed after staining. Staining was performed using oil red O stain. Five dpf larvae were anesthetized with MS-222 (0.04%), fixed in 4% PFA, and kept at 4 °C overnight. For oil red O staining, the fixed zebrafish tissue was dehydrated using a sucrose solution. The slices were fixed for 10 min in 4% paraformaldehyde and dehydrated. Oil red O staining was viewed with a light microscope.

The cholesterol content was determined using the same batch of zebrafish embryos developed to 5 dpf. The grouping was the same as above, and each group had 3 replicates with 10 embryos per replicate. The embryos were sampled, fixed at 9 dpf, and then stained using the Filipin method. Five dpf larvae were anesthetized with MS-222 (0.04%), fixed in 4% PFA, and kept at 4 °C overnight. Nine dpf zebrafish were incubated in 0.05 mg/mL Filipin working solution at room temperature in the dark for 30 min. The samples were rinsed three times with PBST and observed under a fluorescence microscope (Nikon, Tokyo, Japan).

The zebrafish eyes were collected and their lenses were taken out. The eyes were enucleated and fixed in 4% paraformaldehyde for 24 h. After that, the lenses were rinsed with distilled water (1.5 mL per well, 3 times in 1 h), and then isolated lenses with hyaloid-retinal vessels were obtained following incubation with 3% trypsin in Tris-HCl buffer (pH 7.8) for 80 min at 37 °C. A single-photon laser scanning confocal microscope was used to acquire all images (Olympus, Tokyo, Japan). The data were analyzed using the Image J software (Image J 1.52a). The average diameter of the zebrafish eye lens vascular network was calculated. The content of triglycerides was analyzed using the image software and gray scale analysis, but the cholesterol and blood vessels in the stained zebrafish lens structure were quantified by fluorescence analysis.

### 4.5. Effects of T3 on the Lipid Metabolism Pathway in Adult Zebrafish

Normal cultured zebrafish and high-sugar- and high-fat-cultured zebrafish were used as templates for metabolic pathway analysis. The control group (Control), high-glucose and high-fat blank group (HFD), and high-glucose and high-fat T3 group (Tocotrienols) were designed. A total of 25 mg of zebrafish liver tissue was weighed and ground in 800 ul extraction buffer (dichloromethane/methanol = 3:1, *v*/*v*, pre-cooled at −20 °C) for 5 min. After homogenizing the samples for 5 min with a TissueLyser, they were sonicated for 10 min and incubated for 1 h at −20 °C. A solution of isopropanol (IPA)/acetonitrile (ACN)/water (2:1:1, *v*/*v*/*v*) was used to reconstitute the samples before analysis. All samples were mixed in equal amounts to prepare the QC samples. The injection volume of each sample was 5.0 μL. Chromatographic separation was performed using an Acquity UPLC HSS T3 column (2.1 × 100 mm, 1.8 μm, Waters Corp., Milford, CA, USA) using a Waters ACQUITY UPLC system, equipped with a binary solvent delivery system.

### 4.6. Effects of T3 on the Expression of Lipid Metabolism-Related Genes in the Liver of Adult Zebrafish

TRIzol reagent (Takara, Dalian, China) was used to extract the total RNA from the liver in each group according to the protocols. The RNA quality was examined by 1% agarose gel electrophoresis. The RNA was then reverse-transcribed into cDNA using a HiScript II 1st Strand cDNA Synthesis Kit (Vazyme, Nanjing, China). The qPCR experiment was conducted following the SYBR qPCR master mix reagent instructions (Vazyme, Nanjing, China). The expression levels of the *cpt-1a*, *hmgcr*, *pparγ*, and *cyp7a1* genes were detected (primer sequences are provided in Table 1). The cycling conditions were as follows: 30 s of initial denaturation at 95 °C, 40 cycles consisting of 30 s at 95 °C and 30 s at 60 °C, and a final melting curve program. The gene expression levels were calculated by the 2^−ΔΔCt^ method [4].

### 4.7. Statistical Analyses

All results are expressed as the mean ± standard deviation (SD). Statistical assessments were conducted utilizing SPSS (IBM SPSS Statistics, Version 22.0). The data underwent normal distribution testing and variance homogeneity examination using the Kolmogorov–Smirnov and Levene tests. A one-way ANOVA was employed for statistical evaluation, succeeded by the LSD test. A statistical analysis was conducted using a paired t-test, where *p* > 0.05 indicated no significant difference, 0.01 < *p* < 0.05 indicated a significant difference, and *p* < 0.01 represented a highly significant difference. The statistical analyses were performed using GraphPad Prism 5 (GraphPad Prism software, San Diego, CA, USA).

## 5. Conclusions

We developed a zebrafish model of T2MD using a high-sugar and high-fat diet. Through oil red O and Filipin staining, we confirmed that T3 reduced the accumulation of triglycerides and cholesterol in zebrafish to the levels of a normal diet. At the same time, the results of zebrafish eye blood vessels showed that the lipid-lowering effect of T3 could significantly improve the phenotype. Zebrafish non-targeted lipidomics studies showed that T3 reduced fat synthesis in zebrafish by affecting steroid biosynthesis and steroid hormone biosynthesis in zebrafish. In addition, we demonstrated that tocotrienol can inhibit lipogenesis and fat storage by downregulating the expression of Pparγ and Rxrα, and its hypolipidemic and cholesterol-lowering effects were demonstrated in both adult and juvenile zebrafish. Overall, this study provided empirical data to support the potential application of T3 as a feed additive to address excessive lipid accumulation in aquatic organisms.

## Figures and Tables

**Table 1:** Primers used to detect the genes in this study.

Gene Name	Primer F (5′-3′)	Primer R (5′-3′)
*Cpt-1a*	TGCGGTCTTGCACTACAGAG	GTGGACAGTCTCCAAGGCTC
*Hmgcr*	TCGTGGAGTGCCTGGTGATTGGT	TGGGTCTGCCTTCTCTGCTCTCTC
*PPARγ*	TGGAGCCCAAGTTTCAGTTCGC	GTATGAGTTGTGCATGTTCGGTC
*Cyp7a1*	GCTCTACTTCCACCTGATT	ATGTCTTCTGCGTATTCCT
β-actin	TGGAGCCCAAGTTTCAGTTCGC	GTATGAGTTGTGCATGTTCGGTC

## Data Availability

All experimental results will be provided to anyone on request.

## Supplementary Materials

The supporting information can be downloaded at: https://www.mdpi.com/article/10.3390/ijms25052954/s1.
