# Supplementary material for: Hypolipidemic Effect of Rice Bran Oil Extract Tocotrienol in High-Fat Diet-Induced Hyperlipidemia Zebrafish (Danio Rerio) Induced by High-Fat Diet
Source: Int J Mol Sci. 2024 Mar 3;25(5):2954. doi: 10.3390/ijms25052954 (PMC10931685; doi:10.3390/ijms25052954)
Supplement: Supplementary file 1 [file ijms-25-02954-s001.zip › ijms-2854609-supplementary.pdf]

Supplementary materials

The end of the experiment after 96 h

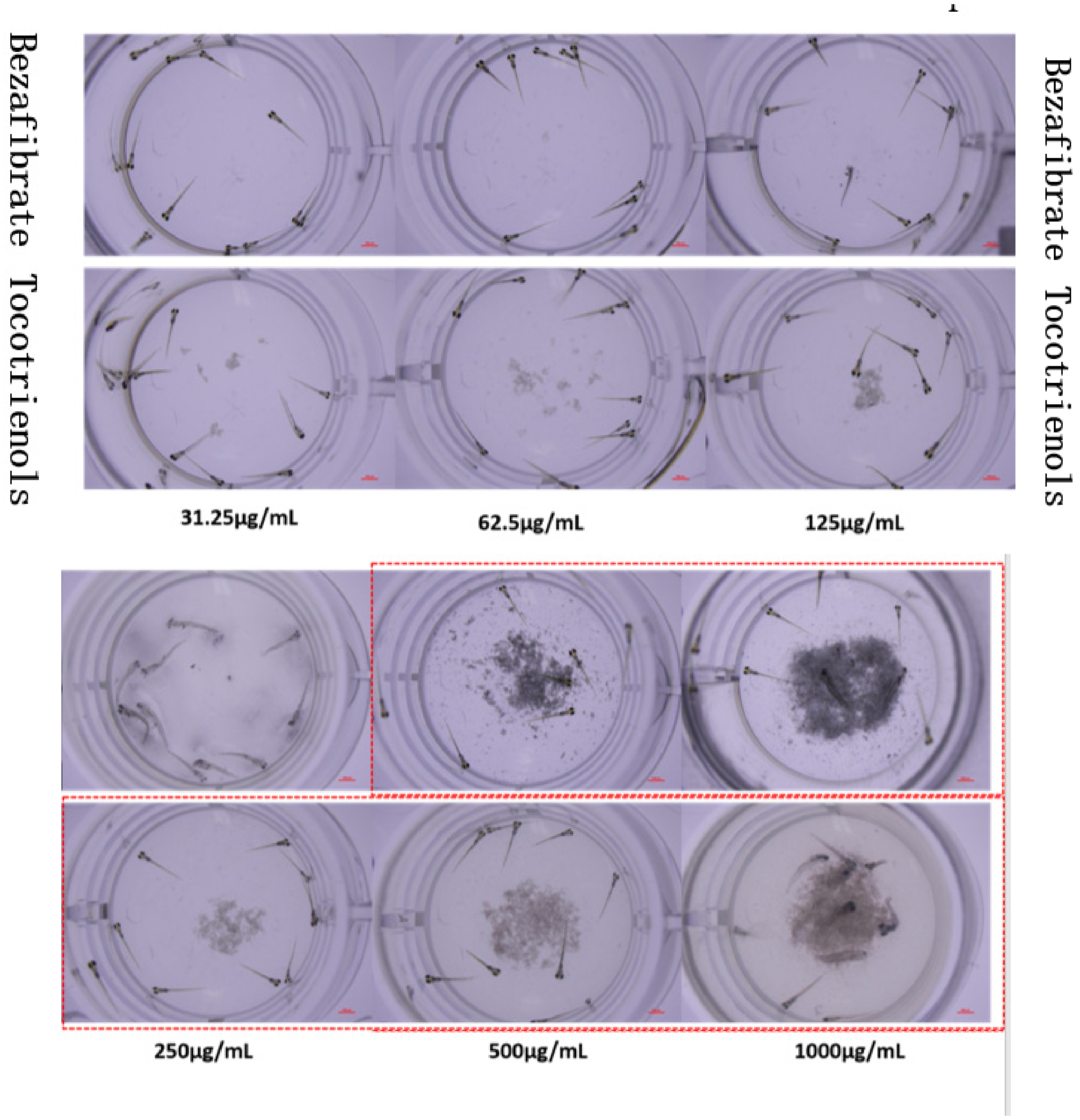

Figure S1. Mortality rates and morphological abnormalities were monitored at 24, 48, 72, and 96 h. No instances of death or deformity were observed in zebrafish subjected to tocotrienol or in the control group.
